# Comparing primary care Interprofessional and non-interprofessional teams on access to care and health services utilization in Ontario, Canada: a retrospective cohort study

**DOI:** 10.1186/s12913-021-06595-x

**Published:** 2021-09-14

**Authors:** Wissam Haj-Ali, Brian Hutchison, Rahim Moineddin, Walter P. Wodchis, Richard H. Glazier

**Affiliations:** 1 Dalla Lana School of Public Health, Toronto, Ontario Canada; 2 Institute of Health Policy, Management and Evaluation, University of Toronto, 155 College Street, Toronto, Ontario M5T 3M6 Canada; 3 Canadian Centre for Health Economics, Toronto, Canada; 4 Institute for Clinical Evaluative Sciences, Toronto, Canada; 5 Departments of Family Medicine and Health Research Methods, Evidence and Impact, McMaster University, Hamilton, Canada; 6 Department of Family and Community Medicine, University of Toronto, Ontario, Canada; 7 Trillium Health Partners, Institute for Better Health, Toronto, Ontario Canada; 8 MAP Centre for Urban Health Solutions, St. Michael’s Hospital, Toronto, Canada

**Keywords:** Primary care, Interprofessional teams, Patient experience, Access, Health services utilization

## Abstract

**Background:**

Many countries, including Canada, have introduced primary care reforms to improve health system functioning and value. The purpose of this study was to examine the association between receiving care from interprofessional primary care teams and after-hours access to care, patient-reported walk-in clinic visits and emergency department use.

**Methods:**

We conducted a retrospective cohort study linking population-based administrative databases to Ontario’s Health Care Experience Survey (HCES) between 2012 and 2018. We adjusted for physician group characteristics as well as individual physician and patient characteristics while assessing the relationship between receiving care from interprofessional teams and the outcomes of interest.

**Results:**

As of March 31st, 2015, there were 465 physician groups with HCES respondents of which 177 (38.0%) were interprofessional teams and 288 (62.0%) were non-interprofessional teams in the same blended capitation reimbursement model. In this period, there were 4518 physicians with HCES respondents, of whom 2131 (47.2%) were in interprofessional teams and 2387 (52.8%) were in non-interprofessional teams. There were 10,102 HCES respondents included in this study, of whom 42.4% were in interprofessional teams and 42.3% were in non-interprofessional teams. After adjustment, we found that being in an interprofessional team was associated with an increase in the odds of patients reporting same/next day access to care by 12.0% (OR = 1.12 CI = 1.00 to 1.24 *p*-value 0.0436) and a decrease in the odds of patients reporting walk-in clinic use by 16% (OR = 0.84 CI = 0.75 to 0.94 *p*-value 0.0019). After adjustment, there were no significant differences in patient-reported after-hours access to care and emergency department use.

**Conclusions:**

Ontario has invested heavily in interprofessional primary care teams. As compared to patients in non-interprofessional teams, patients in interprofessional teams self-reported more timely access to care and less walk-in clinic use but no significant difference in self-reported access to after-hours care or in emergency department use. For jurisdictions aiming to expand physician voluntary participation in interprofessional teams, our study results inform expectations around access to care and health services utilization.

## Background

Moving towards value-based healthcare is a priority for healthcare systems internationally [1]. The pursuit of providing value-based health care revolves around three aims: improving the experience of care, improving the health of populations, and reducing per capita costs [2]. A strong primary care system is recognized as the cornerstone of health systems and is associated with better outcomes, improved patient experience and lower cost [3]. Many countries around the world, including Canada, have introduced primary care reform to deliver on those goals.

During the economic recession in the 1990s, there has been limited investments in primary care innovation in Canada [4]. A decade later, primary care reform initiatives started to emerge in Canada in response to various recommendations from federal and provincial committees [5, 6]. In line with the Canadian healthcare reform movement, Ontario has undergone three major primary care policy initiatives: new physician payment and governance models, enrolment of patients with a primary care physician and support for the development of interprofessional teams [7]. Interprofessional teams are “groups of professionals from different disciplines who communicate and work together in a formal arrangement to care for a patient population in a primary care setting.” [8] They typically include primary care physicians, nurses or nurse practitioners, and at least one other health care professional (e.g. pharmacist, social worker, dietitian or physiotherapist). Interprofessional teams are also eligible for funding an administrator or executive director.

During the last 20 years, more than 30% of primary care physicians have willingly moved from fee-for-service payment model to a blended capitation. Some of those physicians have received extra funding to set up and deliver interprofessional team-based care. Currently, the dominant blended capitation model in Ontario is called Family Health Organization (FHO). FHOs have formal patient enrollment, electronic medical records, physician-led governance and a minimum of three physicians practicing together. They offer comprehensive care, including preventive health care services, chronic disease management and health promotion, through a combination of regular physician office hours and after-hours services. FHOs were eligible to apply for additional funding for allied health professionals to join their practice and become interprofessional primary care teams called Family Health Teams.

The government’s priorities in establishing interprofessional teams were to increase access to primary care and appropriate healthcare services utilization [9]. Physicians in FHO models in Ontario are required to provide after-hours access to care and receive a bonus when their patients do not seek services from physicians outside of their group, such as in walk-in clinics. The bonus is not affected if their patients visit the emergency department. Interprofessional team-based care is thought to free up some of the physicians’ time by delegating tasks to other health care professionals within their scope of practice [10]. Access to quality primary care can reduce the need for unnecessary and more expensive services [11]. Treating less-urgent conditions in primary care could improve continuity of care and patient experience [12, 13].

Several studies conducted in Ontario have compared capitation-based interprofessional teams to other funding and delivery models of care on specific measures of quality [14–20]. However, little research to date has evaluated the association between the interprofessional aspect of primary care teams and access to care and health services utilization. Our study examined the association between receiving care from interprofessional versus non-interprofessional primary care teams and patient-reported timely and after-hours access to care, patient-reported walk-in clinic visits and emergency department use. We hypothesised that interprofessional teams would be better performers on these measures given their enhanced capacity and structure. Evidence from our setting that underwent large-scale primary care reform will be relevant to other jurisdictions contemplating innovations in primary care delivery and, specifically, the adoption of interprofessional team-based primary care.

## Methods

### Setting

Ontario is a province in Canada and had a population on 14.7 million people in 2020 [21]. Permanent residents of Ontario are fully insured for physician primary care services through the Ontario Health Insurance Plan (OHIP) with no co-payment or deductible. Primary care organization and payment models have evolved over the course of the last 18 years. Three dominant practice models exist in Ontario—enhanced fee-for-service, non-team blended capitation and team-based blended capitation. These models are described in detail elsewhere [7, 22, 23].

The focus of this study was on the dominant blended capitation model—FHO—within which physicians practice in either interprofessional or non-interprofessional teams. When patients seek primary care services outside the practice in which they are enrolled, for example in walk-in clinics, the FHO loses a bonus payment equal to the fee-for-service payments to the physician who treated the patient, to a maximum bonus of 18.59% of the practice’s total capitation [24]. There is no deduction if an enrolled patient visits an emergency department for non-emergency care. FHOs are required to provide at least one three-hour block of after-hours services per week for each physician in the group, to a maximum of five three-hour blocks per week for practices with five or more physicians. Contracts define “after-hours” as Monday to Thursday after 5 p.m. or any time on the weekend—that is, any time from Friday after 5 pm through Sunday [25].

### Design and population

**Fig. 1:**
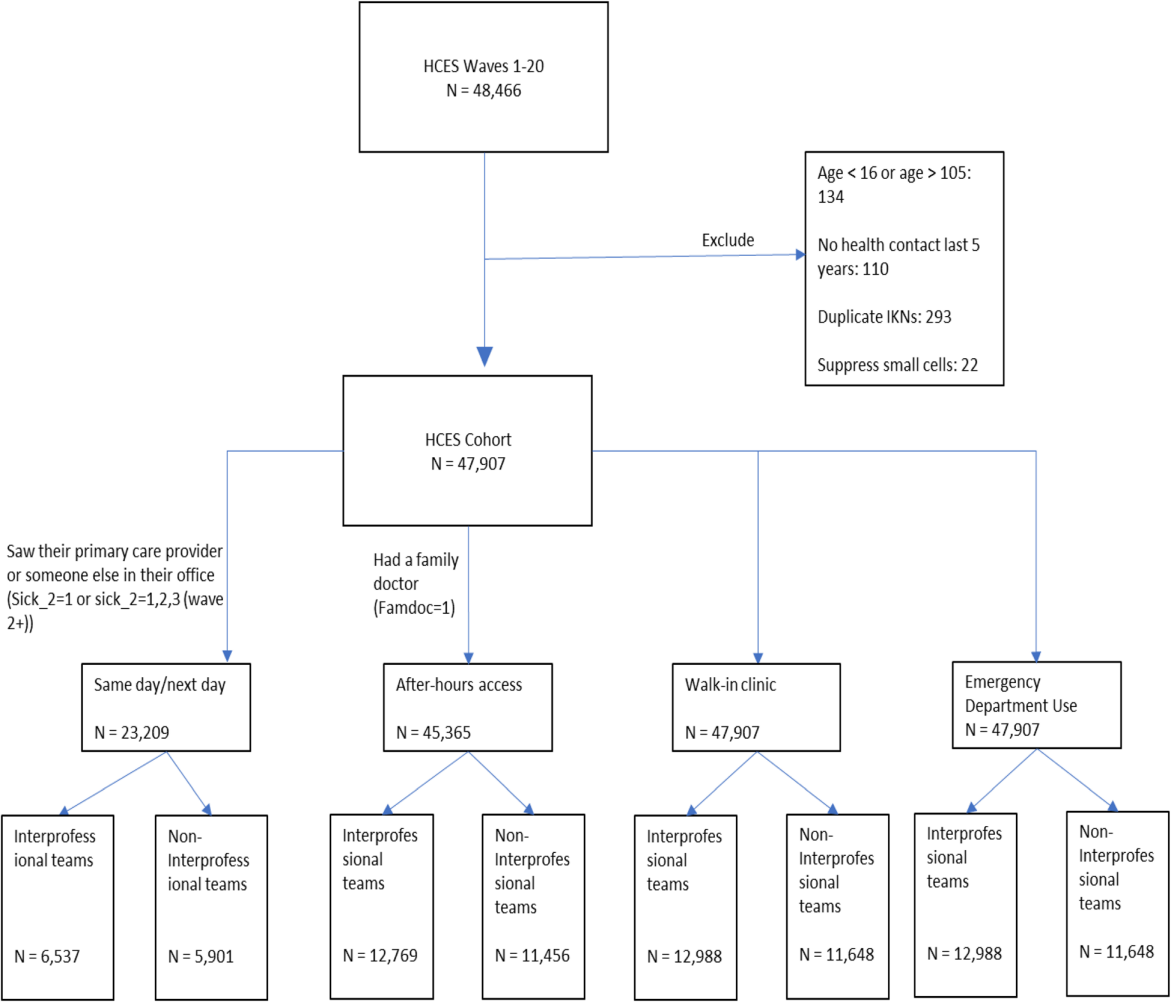
Study population flow diagram

We conducted a retrospective cohort study where we linked several population-based administrative databases to the Health Care Experience Survey (HCES) using encoded identifiers at ICES (formerly known as the Institute for Clinical Evaluative Sciences) to form data extractions and identify the population of interest (Fig. 1). The HCES is collects information to understand Ontarians’ experience in obtaining primary care services and helps the Ministry of Health in planning health care programs and policies. The HCES survey is conducted continuously by the Institute for Social Research (ISR) at York University, with data being provided to the Ontario Ministry of Health every 3 months (termed a ‘wave’).

The study population comprised respondents to the HCES over six fiscal years (April 1 – March 31) from 2012/13 to 2017/18. The HCES targets persons 16 years and older who live in private dwellings in Ontario. People living in institutions, in households without telephones are excluded. The study included respondents from 20 quarterly waves of the HCES that were conducted between October 2012 and October 2017. The average response rate was 51% during that period. Once households were sampled in the HCES, they were removed from the sampling frame for 2 years. Respondents who responded to the survey more than once throughout the study period were excluded.

**Fig. 2:**
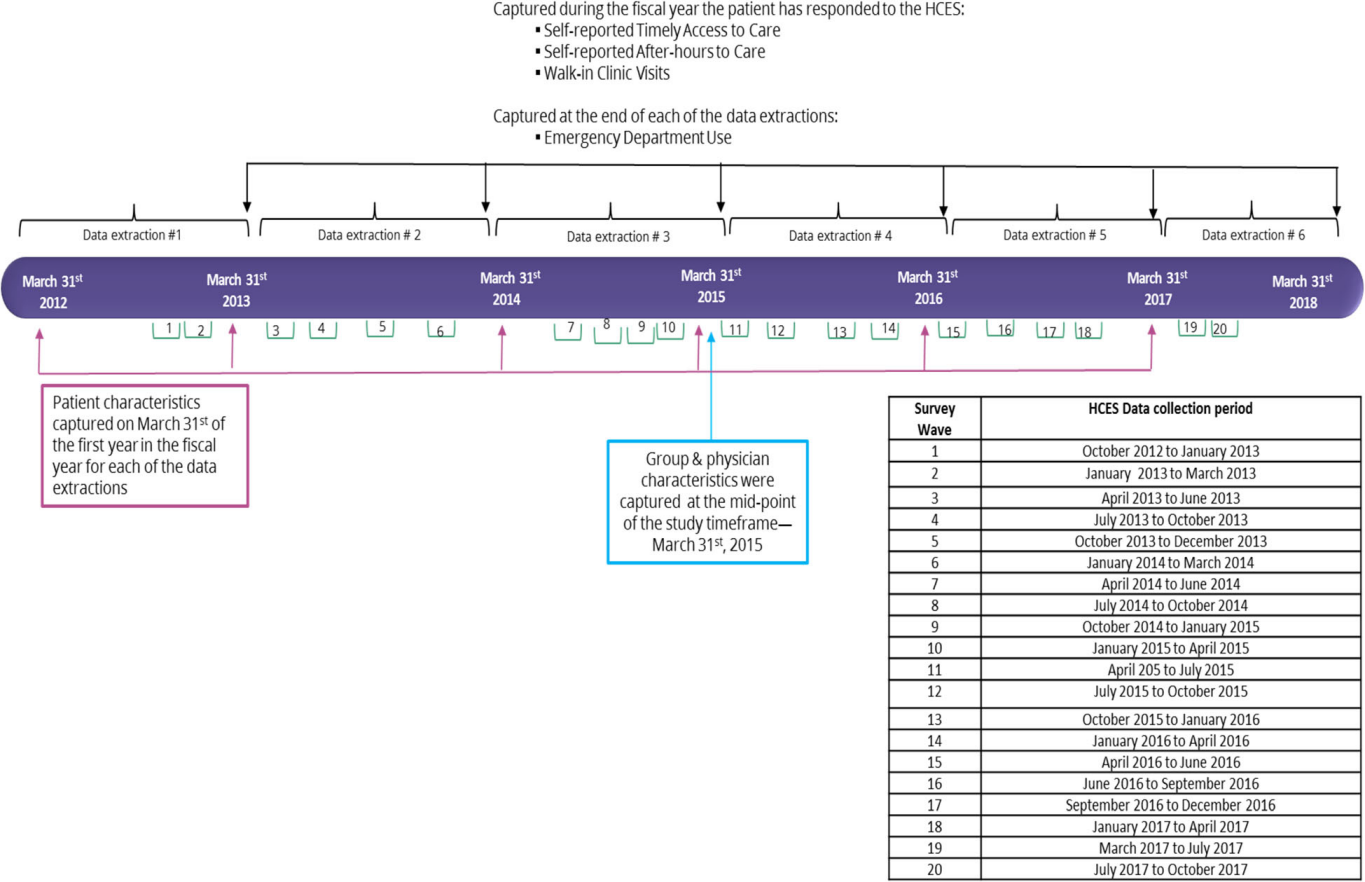
Data extractions and cohort generation

For each of the data extractions, we identified respondent to the HCES at the end of the fiscal year. To be included in the study, respondents had to be consistently in an FHO blended capitation model throughout the observation period for the fiscal year they responded to the HCES. We captured patients’ characteristics at the beginning of the fiscal year they responded to the HCES. Self-reported timely access to care, after-hours access to care and walk-in clinic visits were captured during the fiscal year the patient responded to the HCES and ED visits were captured at the end of that fiscal year from health administrative data. Physician group and physicians’ characteristics were captured at the mid-point of the study timeframe, March 31st, 2015 (Fig. 2).

### Measures and data sources

#### Exposure

Enrolment in a FHO blended capitation model, with an interprofessional team was the exposure. The exposure variable was retrieved from a population and demographics database—the Client Agency Program Enrolment tables that identify the patient enrolment model and the physician with whom patients are enrolled. A separate file provided by the Ontario Ministry of Health (MOH) to ICES identified physicians who are part of an interprofessional team versus a non-interprofessional team.

#### Outcomes

The outcomes included patient-reported timely access to care, patient-reported after-hours access to care, patient-reported walk-in clinic use and emergency department use. Patient-reported timely access to care, after-hours access to care and walk-in clinic use were derived from the HCES (How many days did it take from when you first tried to see your provider to when you actually saw them or someone else in their office? (sick_3); The last time when you needed medical care in the evening, on a weekend, or on a public holiday, how easy or difficult was it to get care without going to the emergency department? (access_5); Have you been to a walk-in clinic because you were sick or for a health-related problem in the 12 months? (wi_1)). The HCES is a quarterly survey of a random sample of the Ontario population, 16 years and older, conducted on behalf of the MOH by the Institute for Social Research at York University. The survey focuses on Ontarians’ primary care experience, including access to care, to generate regional and province-level data. The National Ambulatory Care Reporting System (NACRS) was used to derive emergency department visits.

#### Physicians, physicians groups and patient characteristics

All characteristics were derived from administrative databases available at ICES. Physicians’ characteristics included age, sex, years since graduation, Canadian graduate status and number of years in practice. Physician group characteristics included the number of physicians per group and number of years under the capitation model.

Patient characteristics included age, sex and OHIP registration (as proxy for immigration), neighborhood income quintiles, rurality, and Resource Utilization Bands [26, 27].

#### Analysis

For the descriptive results, we generated counts and percentages for categorical variables and means and standard deviations for continuous variables to describe the characteristics of physician groups and physicians who were either in interprofessional or non-interprofessional teams in relation to the outcomes of interest. For the patient variables, we generated sample weighted descriptive statistics. The probability weights assigned to respondents in the HCES were dependent on the probability of being selected, which was determined from the sampling design.

For the outcomes, we ran sample weighted survey logistic regressions to model each of the outcomes while adjusting for the respective physician group, physician and patient characteristics.

All study analyses were conducted using SAS v.9.3 and statistical significance was assessed at a *p*-value < 0.05.

## Results

### Baseline group, physician and patient characteristics comparing HCES respondents in interprofessional teams versus non-interprofessional teams

As of March 31st, 2015, there were 465 FHO physician groups with HCES respondents of which 177 (38%) were interprofessional teams and 288 (62%) were non-interprofessional teams. Interprofessional teams with HCES respondents had more physicians per group as compared to non-interprofessional teams (means = 13.1 versus 8.84, respectively) and more years under the capitation model (means = 6.0 versus 4.3 respectively).

In this period, there were 4518 FHO physicians with HCES respondents of whom 2131 (47.2%) were practicing in interprofessional teams and 2387 (52.8%) were practicing in non-interprofessional teams. Interprofessional teams compared to non-interprofessional team physicians had: fewer patients per physician (mean = 1366 versus 1555, respectively); more female physicians (46.3% versus 43.8%, respectively); more physicians in the younger age group under 40 years old (15.4% versus 9.3%, respectively); more physicians who were Canadian graduates (80.9% versus 74.4%, respectively); fewer years in practice (29.1% versus 17.6%, respectively in the 5 to 15 years category) (Table 1).

**Table 1:** Physician Group and physicians characteristics (on March 31st, 2015) – comparing HCES respondents in interprofessional teams to respondents in non-interprofessional teams

	Interprofessional Teams		Non-interprofessional teams	
**Physician Group characteristics**				
**Physician Groups No. (%)**	177	38.0	288	62.0
**Number of physicians per group, Mean (SD)**	13.1	10.7	8.8	7.6
**Years under the capitation model, Mean (SD)**	6.0	3.0	4.2	2.6
**Physicians characteristics**				
**Physicians No. (%)**	2131	47.2	2387	52.8
**Number of patients per physician, Mean (SD)**	1366	615.1	1555	665.2
**Sex No. (%)**				
Female	987	46.3	1045	43.8
**Age group in Yrs. No. (%)**				
< 40	329	15.4	222	9.3
40–64	1417	66.5	1607	67.3
> 64	358	16.8	534	22.4
Missing	27	1.3	24	1.0
**Country of medical graduation Canada No. (%)**				
Yes	1724	80.9	1775	74.4
**Years in practice No. (%)**				
< 5	47	2.2	41	1.7
5_15	620	29.1	420	17.6
16–25	495	23.2	606	25.4
> 25	969	45.5	1320	55.3
Missing	0	0	0	0

There were 10,102 HCES respondents included in this study of whom 42.4% were in interprofessional teams and 42.3% were in non-interprofessional teams. Interprofessional as compared to non-interprofessional teams had fewer HCES respondents who were immigrants (3.1% versus 5.1%, respectively); fewer HCES respondents in the highest income quintile (23.3% versus 26.4%, respectively); more HCES respondents residing in rural areas (14.2% versus 5.8%, respectively) and fewer patients with two or more comorbidities (42.6% versus 44.3%, respectively) (Table 2).

**Table 2:** Patients’ characteristics comparing HCES respondents in interprofessional teams to respondents in non-interprofessional teams in the year they responded to the survey

	Interprofessional Teams		Non-interprofessional Teams	
**Patients total**	12,988	52.7	11,648	47.3
**Sex No. (%)**				
Female	7678	57.6	6856	57.7
**Age group, yr. No. (%)**				
16–44	3819	33.0	3653	34.9
45–64	5272	42.4	4661	41.4
65–84	3602	23.1	3071	22.1
84+	295	1.5	263	1.6
Missing	0	0	0	0
**New OHIP registrants (within 10 years) No. (%)**	355	3.1	460	5.1
**Income quintile, No. (%)**				
1 (low)	2089	13.8	1764	13.9
2	2468	18.6	2228	17.9
3	2697	21.2	2295	19.6
4	2822	22.8	2550	22.0
5 (high)	2888	23.3	2784	26.4
Missing	24	0.3	27	0.2
**Rurality Index of Ontario, No. (%)**				
Largest Urban (0)	3759	33.6	4000	42.6
Large urban (1 to 9)	2388	17.1	4078	29.4
Small-urban (10 to 39)	4823	34.2	2737	21.7
Rural (≥40)	1892	14.4	763	5.8
Missing	126	0.7	70	0.4
**Resource utilization band (RUB), No. (%)**				
1	629	5.4	471	4.3
2	2128	17.7	1802	16.5
3	6746	51.0	6417	54.6
4	2031	15.0	1869	15.4
5 (very high user)	823	5.4	674	5.1
Non-user and Missing	631	5.5	415	4.2
**Patients with Chronic disease**				
2 + Co-morbidity No. (%)	6096	42.6	5628	44.3
3+ comorbidities No. (%)	3482	23.3	3207	24.5
4+ comorbidities No. (%)	1828	11.9	2686	12.4
5+ comorbidities No. (%)	894	5.8	791	6.1

### Univariate analysis

#### Patient-reported timely access to care and after-hours access to care comparing HCES respondents in interprofessional teams versus non-interprofessional teams

HCES respondents in interprofessional teams were slightly more likely to report timely access to care (same/next day) when compared to patients in non-interprofessional teams (39.9% versus 39.1%). HCES respondents in interprofessional teams were less likely to report easy or somewhat easy access to after-hours care compared to patients in non-interprofessional teams (30.8% versus 35.2%). The results stratified by physicians charateristics are presented in Tables 3 and 4. Tables 5 and 6 present the results stratified by patient characteristics.

**Table 3:** Patient-reported timely access to care (same/next day) in the year patients responded to the HCES by physicians’ characteristics identified on March 31st, 2015

	Interprofessional Teams		Non-interprofessional Teams	
	Denominator	Percentage	Denominator	Percentage
**Physicians characteristics**				
**Sex**				
Female	2621	39.5	2256	35.5
Male	3880	39.3	3614	37.4
Missing	36	19.4	31	29.0
**Age group**				
< 40	761	40.6	433	33.5
40–64	4381	39.1	3973	35.9
> 64	1243	40.3	1369	40.6
Missing	152	27.6	126	25.4
**Country of medical graduation Canada**				
No	1176	36.6	1318	35.9
Yes	5209	40.2	4457	37.2
Missing	152	27.6	126	25.4
**Years in practice**				
< 5	151	35.1	110	30.0
5_15	1553	40.5	892	33.0
16–25	1469	35.5	1483	34.1
> 25	3328	40.7	3385	39.0
Missing	36	19.4	31	29.0

**Table 4:** Patient-reported after-hours access to care (very easy and somewhat easy) in the year patients responded to the HCES by physicians’ characteristics identified on March 31st, 2015

	Interprofessional Teams		Non-interprofessional Teams	
	Denominator	Percentage	Denominator	Percentage
**Physicians characteristics**				
**Sex**				
Female	4917	32.3	4246	34.5
Male	7769	29.9	7145	34.0
Missing	83	27.7	65	29.2
**Age group**				
< 40	1385	31.2	829	36.4
40–64	8542	31.3	7605	33.9
> 64	2523	29.4	2791	34.6
Missing	319	26.6	231	29.0
**Country of medical graduation Canada**				
No	2324	27.6	2572	33.7
Yes	10,126	31.6	8653	34.4
Missing	319	26.6	231	29.0
**Years in practice**				
< 5	285	27.0	205	34.1
5–15	2907	31.9	1679	33.7
16–25	2865	31.7	2791	33.0
> 25	6629	30.1	6716	34.8
Missing	83	27.7	65	29.2

**Table 5:** Patient-reported timely access to care (same/next day) by patients’ characteristics identified at the year they have responded to the HCES

	Interprofessional Teams		Non-interprofessional Teams	
	Denominator	Weighted Percentage	Denominator	Weighted Percentage
**Overall self-reported timely Access to care**	6537	39.9	5901	39.1
**Sex**				
Female	4159	40.5	3681	39.6
Males	2378	38.8	2220	38.2
Missing	0	–	0	–
**Age group, yr.**				
16–44	1964	41.0	1840	38.1
45–64	2781	36.8	2467	38.1
65+	1680	44.5	1479	42.5
Missing	112	40.0	115	43.1
**New OHIP registrants (within 10 years)**				
No	6351	39.9	5659	38.6
Yes	180	36.7	238	47.1
**Income quintile**				
1 (low)	1030	37.6	862	35.8
2	1239	39.7	1132	40.3
3	1340	41.1	1193	37.1
4	1419	38.9	1254	37.9
5 (high)	1500	41.2	1445	42.2
Missing	9	29.3	15	32.9
**Rurality Index of Ontario**				
Largest Urban (0)	2010	42.8	2133	42.9
Large urban (1 to 9)	1276	42.2	2077	37.1
Small-urban (10 to 39)	2375	39.1	1312	36.1
Rural (≥40)	832	30.1	345	29.5
Missing	44	18.7	34	31.3
**Resource utilization band (RUB)**				
1	234	42.1	167	42.0
2	868	38.4	700	36.7
3	3625	39.5	3421	39.3
4	1172	42.6	1114	39.2
5 (very high user)	508	40.5	425	41.7
Non-user and Missing	130	44.4	74	24.1
**Patients with Chronic disease**				
2 + Co-morbidity				
No	3221	38.2	2817	37.8
Yes	3316	41.8	3084	40.4
3+ comorbidities				
No	4602	40.0	4087	37.8
Yes	1935	39.4	1814	42.6
4+ comorbidities				
No	5505	39.7	4931	38.6
Yes	1032	41.1	970	41.9
5+ comorbidities				
No	6022	39.8	5444	38.8
Yes	515	40.8	457	43.1

**Table 6:** Patient-reported after-hours to care (very easy and somewhat easy) by patients’ characteristics identified in the year they have responded to the HCES

	Interprofessional Teams		HCES Respondents in Non-interprofessional Teams	
	Denominator	Weighted Percentage	Denominator	Weighted Percentage
**Overall patient-reported after-hours access to care**	12,769	30.8	11,456	35.2
**Patients characteristics**				
**Sex**				
Female	7584	33.4	6765	37.0
Males	5185	30.9	4691	32.8
Missing				
**Age group, yr.**				
16–44	3703	38.9	3544	39.3
45–64	5199	30.9	4602	34.2
65+	3575	26.0	3051	31.1
Missing	292	28.5	259	33.9
**New OHIP registrants (within 10 years)**				
Yes	346	30.4	445	40.0
NO	12,410	32.4	10,997	35.0
Missing	13	42.8	14	47.9
**Income quintile**				
1 (low)	2038	32.3	1718	34.5
2	2427	29.6	2187	33.8
3	2655	32.1	2268	33.2
4	2777	34.7	2511	35.6
5 (high)	2849	32.4	2745	37.8
Missing	23	44.4	27	33.2
**Rurality Index of Ontario**				
Largest Urban (0)	3700	38.3	3931	37.8
Large urban (1 to 9)	2344	41.5	4010	39.0
Small-urban (10 to 39)	4752	28.0	2699	28.6
Rural (≥40)	1852	18.4	746	23.2
Missing	121	23.4	70	24.6
**Resource utilization band (RUB)**				
1	609	33.4	457	38.4
2	2073	35.8	1771	37.9
3	6671	30.9	6334	35.1
4	2013	32.0	1845	34.4
5	816	30.8	671	33.7
Non-user and Missing	587	39.6	378	37.7
**Patients with Chronic disease**				
2 + Co-morbidity				
No	6732	34.0	5875	36.1
Yes	6037	30.1	5581	34.2
3+ comorbidities				
No	9322	33.2	8274	35.3
Yes	3447	29.4	3182	35.0
4+ comorbidities				
No	10,963	32.6	9784	35.2
Yes	1806	30.2	1672	35.7
5+ comorbidities				
No	11,886	32.5	10,672	35.0
Yes	883	29.8	784	38.4

#### Patient-reported walk-in clinic visits and emergency department use comparing HCES respondents in interprofessional teams versus non-interprofessional teams

HCES respondents in interprofessional teams reported a lower percent of walk-in clinic visits compared to patients in non-interprofessional teams (19.7% versus 28.2%, respectively) (Table 7). A higher percent of HCES respondents in interprofessional teams had emergency department visits as compared to patients in non-interprofessional teams (26.7% versus 23.5%, respectively) (Table 8). The results stratified by physician charateristics are presented in Tables 9 and 10.

**Table 7:** Patient-reported walk-in clinic by patients’ characteristics identified at the year they have responded to the HCES

	Interprofessional Teams		Non-Interprofessional Teams	
	Denominator	Weighted Percentage	Denominator	Weighted Percentage
**Overall patient-reported walk-in clinic**	12,988	19.7	11,648	28.2
**Patients characteristics**				
**Sex**				
Males	5310	17.7	4792	26.1
Female	7678	21.2	6856	29.7
Missing	0	–	0	–
**Age group, yr.**				
16–44	3819	29.5	3653	37.6
45–64	5272	17.1	4661	27.4
65–84	3602	11.3	3071	15.9
85+	295	10.1	263	14.9
Missing	0	–	0	–
**New OHIP registrants (within 10 years)**				
Yes	355	23.6	460	34.2
No	12,620	19.6	11,174	27.8
Missing	13	21.0	14	40.8
**Income quintile**				
1 (low)	2089	19.2	1764	27.1
2	2468	17.4	2228	27.6
3	2697	20.6	2295	28.7
4	2822	20.4	2550	30.4
5 (high)	2888	20.4	2784	26.8
Missing	24	12.6	27	36.3
**Rurality Index of Ontario**				
Largest Urban (0)	3759	21.8	4000	30.2
Large urban (1 to 9)	2388	32.0	4078	34.8
Small-urban (10 to 39)	4823	16.2	2737	19.8
Rural (≥40)	1892	9.3	763	10.9
Missing	126	11.2	70	34.9
**Resource utilization band (RUB)**				
1	629	18.5	471	26.8
2	2128	17.4	1802	27.8
3	6746	20.2	6417	29.6
4	2031	23.0	1869	30.9
5	823	18.2	674	20.6
Non-user and Missing	631	18.5	415	25.7
**Patients with Chronic disease**				
2 + Co-morbidity				
Yes	6096	17.5	5628	25.9
No	6892	21.4	6020	30.0
3+ comorbidities				
Yes	3482	16.8	3207	24.7
No	9506	20.6	8441	29.3
4+ comorbidities				
Yes	1828	17.0	1686	22.7
No	11,160	20.1	9962	28.9
5+ comorbidities				
Yes	894	17.3	791	20.1
No	12,094	19.9	10,857	28.7

**Table 8:** All ED visits by patients’ characteristics identified in the year they responded to the HCES

	HCES Respondents in Interprofessional Teams		HCES Respondents in Non-interprofessional Teams	
	≥1 ED visits		≥1 ED visits	
	Denominator	Weighted Percentage	Denominator	Weighted Percentage
**Overall ED visits**	12,988	26.7	11,648	23.5
**Sex**				
Males	5310	26.7	4792	22.9
Female	7678	26.7	6856	23.9
Missing	0	–	0	–
**Age group, yr.**				
16–44	3819	26.8	3653	22.3
45–64	5272	24.1	4661	21.8
65–84	3602	29.2	3071	26.1
85+	295	40.0	263	38.4
Missing	0	–	0	–
**New OHIP registrants (within 10 years)**				
Yes	355	20.3	460	22.0
No	12,620	26.9	11,174	23.6
**Income quintile**				
1 (low)	2089	33.3	1764	27.7
2	D/S	D/S	D/S	D/S
3	2697	26.4	2295	23.8
4	2822	24.8	2550	21.9
5 (high)	2888	22.6	2784	21.6
Missing	D/S	D/S	D/S	D/S
**Rurality Index of Ontario**				
Largest Urban (0)	3759	23.5	4000	20.9
Large urban (1 to 9)	2388	22.0	4078	20.3
Small-urban (10 to 39)	4823	27.8	2737	28.0
Rural (≥40)	1892	35.3	763	37.5
Missing	126	38.1	70	30.0
**Resource utilization band (RUB)**				
1	629	19.9	471	15.7
2	2128	19.2	1802	15.2
3	6746	25.7	6417	22.7
4	2031	34.6	1869	31.7
5 (very high user)	823	48.5	674	42.7
Non-user and Missing	631	15.5	415	13.5
**Patients with Chronic disease**				
2 + Co-morbidity				
Yes	6096	32.0	5628	23.5
No	6892	22.0	6020	23.5
3+ comorbidities				
Yes	3482	36.4	3207	32.6
No	9506	23.1	8441	20.0
4+ comorbidities				
Yes	1828	40.6	1686	37.9
No	11,160	24.4	9962	21.0
5+ comorbidities				
Yes	894	47.0	791	41.6
No	12,094	25.2	10,857	22.2

*D/S* Data suppressed where counts are between 1 and 5; additional suppression may be applied where counts are greater than 5 to prevent residual disclosure of suppressed values—in compliant with the Personal Health Information Protection Act (PHIPA) privacy legislation

**Table 9:** Patient-reported walk-in clinic use in the year patients responded to the HCES by physicians’ characteristics identified on March 31st, 2015

	Interprofessional Teams		Non-interprofessional Teams	
	Denominator	Percentage	Denominator	Percentage
**Physicians characteristics**				
**Sex**				
Male	7909	17.3	7279	26.1
Female	4994	20.3	4302	28.8
Missing	85	17.6	67	23.9
**Age group**				
< 40	1418	19.3	842	27.8
40–64	8670	18.7	7717	26.8
> 64	2573	17.4	2852	28.1
Missing	327	16.5	237	20.7
**Country of medical graduation Canada**				
Yes	10,286	18.2	8771	26.3
No	2375	19.8	2640	30.3
Missing	327	16.5	237	20.7
**Years in practice**				
< 5	294	17.3	210	20.5
5_15	2971	19.1	1703	27.2
16–25	2903	19.7	2835	26.7
> 25	6735	17.7	6833	27.4
Missing	85	17.6	67	23.9

**Table 10:** All Emergency Department (ED) visits in the year patients responded to the HCES by physicians’ characteristics identified on March 31st 2015

	Interprofessional Teams		Non-interprofessional Teams	
	≥1 ED visits		≥1 ED visits	
	Denominator	Percentage	Denominator	Percentage
**Physicians characteristics**				
**Sex**				
Male	7909	27.8	7279	24.4
Female	4994	24.8	4302	21.9
Missing	85	29.4	67	20.9
**Age group**				
< 40	1418	26.0	842	27.4
40–64	8670	26.4	7717	22.7
> 64	2573	27.6	2852	24.7
Missing	327	30.9	237	19.8
**Country of medical graduation Canada**				
Yes	10,286	26.1	8771	23.2
No	2375	28.6	2640	24.9
Missing	327	30.9	237	19.8
**Years in practice**				
< 5	294	31.6	210	20.0
5_15	2971	26.4	1703	25.3
16–25	2903	25.5	2835	22.9
> 25	6735	27.1	6833	23.4
Missing	85	29.4	67	20.9

### Multivariate analysis

#### Association between enrollment in an interprofessional team and the outcomes

When we examined timely access to care while adjusting for physician group, physician and patient characteristics, we found that being in an interprofessional team was associated with an increased odd of patient-reported timely (same/next day) access to care of 12% (OR = 1.12 CI = 1.00 to 1.24 *p*-value 0.0436) and decreased odds of self-reporting walk-in clinic use of 16% (OR = 0.84 CI = 0.75 to 0.94 *p*-value 0.0019). We did not find significant differences after adjustment between interprofessional and non-interprofessional teams in patient-reported after-hours access to care or in emergency department use (Table 11).

**Table 11:** Association between enrolment in an interprofessional team-based model and timely access, after-hours access to care, walk-in clinic use and emergency department visits in the year responded to the survey

	**Timely access to care Reference: non-interprofessional teams**			
	**OR**	**95% CI**		***P*-Value**
Unadjusted (null model)	1.03	0.91	1.15	0.6764
†**Adjusted for:**				
Physician group characteristics	1.01	0.90	1.13	0.8397
Group and physicians’ characteristics	1.02	0.92	1.14	0.7041
Physician group, physician and patients	1.12	1.00	1.24	0.0436*
	**After-hours care at the year responded to the survey** **Reference: non-interprofessional teams**			
	**OR**	**95% CI**		***P*-Value**
Unadjusted (null model)	0.87	0.79	0.96	0.0068*
†**Adjusted for:**				
Physician group characteristics	0.81	0.73	0.89	< 0.0001*
Group and physicians’ characteristics	0.81	0.73	0.90	< 0.0001*
Physician group, physician and patients	1.01	0.91	1.12	0.8251
	**Walk-in clinic visits at the year responded to the survey** **Reference: non-interprofessional teams**			
	**OR**	**95% CI**		***P*-Value**
Unadjusted (null model)	0.63	0.57	0.69	< 0.001*
†**Adjusted for:**				
Physician group characteristics	0.67	0.60	0.74	< 0.001*
Group and physicians’ characteristics	0.68	0.61	0.76	< 0.001*
Physician group, physician and patients	0.84	0.75	0.94	0.0019*
	**Emergency department uses at the year responded to the survey** **Reference: non-interprofessional teams**			
	**OR**	**95% CI**		***P*-Value**
Unadjusted (null model)	1.17	1.08	1.28	< 0.0002*
†**Adjusted for:**				
Physician group characteristics	1.20	1.10	1.31	< 0.001*
Group and physicians’ characteristics	1.20	1.10	1.30	< 0.001*
Physician group, physician and patients	1.05	0.95	1.15	0.3234

*p-value significant < 0.05

† Adjustment used physician groups and physicians’ characteristics identified on March 31st, 2015 and patients’ characteristics at the year they have responded to the HCES

When we stratified the analyses by sex and by rurality, we did not find a consistent pattern across the outcomes when comparing interprofessional teams with non-interprofessional teams (results not included but can be made available upon request).

## Discussion

We linked the HCES to administrative databases to examine the association between receiving care from interprofessional primary care teams and patient-reported timely access and after-hours access to care, patient-reported use of walk-in clinics and emergency department use. We found that HCES respondents receiving care from interprofessional teams self-reported more timely access to care and less walk-in clinic use. We did not find a significant difference in patient-reported after-hours access to care or in emergency department visits.

The professional management and clinical structure available through interprofessional teams, such as having an Executive Director and allied health professionals can theoretically support access to care.

Although more timely access to care among patients in interprofessional teams is not an expectation in the contractual agreement between teams and the Ministry of Health, previous evidence indicates that enhanced interprofessional team structure can support the availability of the primary care provider by shifting some of their duties to other team members [28–33]. The evaluations of Patient-Centered Medical Homes in the United States related to timely access to care suggest that greater availability of providers can free more of their time for patient encounters [34]. Our findings of generally low timely access to care are comparable to other reports that found only 43% of Canadians report that they were able to have same- or next-day appointment at their regular place of care and identified that Canada continues to perform below the average on timely access to care when compared to other counties included in the Commonwealth Fund International Health Surveys [24].

Our findings showed a non-significant difference in patient-reported after-hours access to care between interprofessional and non-interprofessional teams. The provision of after-hours care is an expectation that all FHOs need to meet as part of their contractual agreement with the Ministry of Health [32]. Although some interprofessional teams operate out of multiple locations, the after-hours services only need to be offered at one location, which may not be convenient for many of the enrolled patients. Also, only one physician is required to be available during each after-hours block which might not be sufficient evening and weekend availability to meet patients’ needs. Previous evidence that compared a slightly different after-hours access to care measure (asking if respondents providers have an after-hours clinic as opposed how easy or difficult was it to get care without going to the emergency department) found that respondents in interprofessional teams self-reported more after-hours access to care [18].

Although both interprofessional and non-interprofessional teams get penalised equally if their patients visit a walk-in clinic, our finding of significantly lower patient-reported walk-in clinic visits by HCES respondent among interprofessional teams may be explained by the higher patient-reported timely access to care in interprofessional teams, which can contribute to the lower walk-in clinic use. Patients may be less likely to seek care elsewhere if their provider is accessible to them in a timely manner. Additionally, the enhanced administrative structure of interprofessional teams can support reinforcing to patients the need to refrain from walk-in visits as part of being on the group roster. Our findings of a non-significant difference in emergency department use between interprofessional and non-interprofessional teams is consistent with evidence from Canada that looked at utilization in relation to interprofessional team-based care and found differences in quality but not in healthcare utilization [19, 20, 35, 36].

Some of our findings are not fully consistent with an Ontario provincial analysis where throughout the investigated years (2014 to 2017) timely access to care ranged between 44.3 and 39.9% (compared to 39.5% in our study population), easy or somewhat easy after-hours access to care ranged between 48.0 and 46.0% (vs. 33% in our sample) and walk-in clinic use ranged between 29.6 and 30.5% (vs. 24% in our study) [37]. Those differences can be explained by the slightly different timeframe, inclusion of respondents from all primary care models and slightly larger sample that includes people who declined to have their data linked (6%) for the provincial analysis. Additionally, for the timely access to care question, the provincial analysis included respondents with and without a family doctor whereas our study includes only respondents with a family doctor. Through a personal communication with the Ministry of Health representative who is responsible for the survey, we have confirmed that our study results can be mainly explained by those differences.

Interprofessional teams in Ontario had access to several quality improvement initiatives that hypothetically can contribute to improved outcomes over non-interprofessional teams. The Association of Family Health Teams of Ontario through an initiative called Data to Decisions (D2D) supported interprofessional teams in informing quality improvement through performance measurement. D2D was made possible through the investment in more than 30 Quality Improvement Decision Support Specialists (QIDS Specialists) across Ontario to help interprofessional teams to access and use better data to improve care [38]. Timely access to care and emergency department use were among the measurement areas monitored through this initiative [39]. The Quality Improvement and Innovation Partnership (QIIP) was another province wide quality-improvement program implemented between 2008 and 2010 to support interprofessional teams to improve the care they provide [40]. The learning collaboratives used the Institute for Healthcare Improvement’s Breakthrough Series learning model and interprofessional teams were provided with a quality improvement coach who supported and mentored participants throughout the program [41]. Improved access to care was one of the supported quality improvement areas through QIIP [9]. Those investments should theoretically be reflected in better outcomes among interprofessional teams. The government’s first priority in establishing interprofessional teams was to increase access to primary care and health services utilization [32]. Our results show that interprofessional teams perform better than non-interprofessional teams in some but not all aspects related to access to care and health services utilization.

Our study has limitations. First, this is an observational study that cannot address causation. It is also cross-sectional so it is not possible to distinguish whether the outcomes examined were pre-existing or were the result of joining or not joining an interprofessional team. Self-reported timely and after-hours access to care are subject to limitations as measures of performance, respondent recall bias being one of them. People living in institutions, people with non-residential phone numbers, and people with invalid/missing household addresses in the Registered Persons Database (RPDB) are not captured in the HCES. Respondents who were unable to speak English or French or were not healthy enough (physically or mentally) to complete the interview were not surveyed. Second, there are other unmeasured factors that might contribute to the decision of having a walk-in clinic visit or using the emergency department that this study cannot capture. These could include personal preference or judgment during the time the service was needed. Third, access to care can be measured in many different ways. The access questions we investigated in this study provide a specific perspective restricted to timely and after-hours access to care. Previous evidence suggests that different measures of timely access are needed to understand health care system performance.^50^ Fourth, joining interprofessional team-based care was voluntary and our findings could be influenced by some unmeasured factors for physicians who chose to join this model of primary care delivery. Fifth, team composition in terms of allied healthcare professional was not available through administrative databased. Nonetheless, we aimed to capture all measured factors that can be traced through administrative databases. Finally, administrative databases have not been originally collected for research purposes, which presents a limitation in generating and interpreting the information. However, all the databases used for deriving the emergency department measure used in this study have been validated in the Ontario context.

## Conclusion

Ontario has made a major investment in interprofessional team-based care. As compared to patients in non-interprofessional teams, patients in interprofessional teams self-reported more timely access to care and less walk-in clinic use but there was no significant difference in self-reported access to after hours to care and in emergency department use. Our findings can inform other jurisdictions aiming to expand voluntary participation in interprofessional team-based primary care regarding expectations about the relationship between primary care policy, organization and delivery and patient experience and health services utilization. Careful consideration should be given to contractual and policy levers that can incentivise interprofessional team-based care in delivering on intended outcomes such as improving health services utilization.

## Data Availability

The dataset from this study is held securely in coded form at ICES. While data sharing agreements prohibit ICES from making the dataset publicly available, access may be granted to those who meet pre-specified criteria for confidential access, available at www.ices.on.ca/DAS. The full dataset creation plan and underlying analytic code are available from the authors upon request, understanding that the computer programs may rely upon coding templates or macros that are unique to ICES and are therefore either inaccessible or may require modification.

## Abbreviations

HCES: Health care experience survey
FHO: Family health organization
OHIP: Ontario health insurance plan
MOH: Ministry of health
NACRS: National ambulatory care reporting system
RIO: Rurality index of ontario
QIDS: Quality improvement decision support specialists
QIIP: Quality improvement and innovation partnership
RPDB: Registered persons database
